# Patients with Cervical Cancer with and without HIV Infection Have Unique T-cell Activation Profiles despite Similar Survival Outcomes after Chemoradiation

**DOI:** 10.1158/2767-9764.CRC-24-0364

**Published:** 2025-04-14

**Authors:** Emily C. MacDuffie, Luis Cocka, Xiang Lin, Memory Bvochora-Nsingo, Sebathu Chiyapo, Dawn Balang, Bokang Maswabi, Kebatshabile Ngoni, Doreen Ramogola-Masire, Nicola M. Zetola, Zhi Wei, Hao Shen, Sheynaz Bassa, Surbhi Grover, Erle S. Robertson

**Affiliations:** 1 Department of Radiation Oncology, Perelman School of Medicine at the University of Pennsylvania, Philadelphia, Pennsylvania.; 2 Department of Microbiology, Perelman School of Medicine at the University of Pennsylvania, Philadelphia, Pennsylvania.; 3 Department of Computer Science, New Jersey Institute of Technology, Newark, New Jersey.; 4 Department of Oncology, Gaborone Private Hospital, Gaborone, Botswana.; 5 Department of Oncology, Sir Ketumile Masire Teaching Hospital, Gaborone, Botswana.; 6 School of Medicine, University of Botswana, Gaborone, Botswana.; 7 Botswana-UPenn Partnership, Gaborone, Botswana.; 8 Department of Obstetrics and Gynecology, Pennsylvania Hospital, University of Pennsylvania, Philadelphia, Pennsylvania.; 9 Department of Obstetrics and Gynecology, University of Botswana, Gaborone, Botswana.; 10 Department of Radiation Oncology, University of Pretoria, Pretoria, South Africa.; 11 Department of Otorhinolaryngology-Head and Neck Surgery, Abramson Cancer Center, Perelman School of Medicine at the University of Pennsylvania, Philadelphia, Pennsylvania.; 12 Department of Microbiology, Abramson Cancer Center, Perelman School of Medicine at the University of Pennsylvania, Philadelphia, Pennsylvania.

## Abstract

The global burden of cervical cancer is highest in low- and middle-income countries. Women living with human immunodeficiency virus (HIV) infection are particularly affected by cervical cancer despite availability and adherence to antiretroviral therapy. Immune profile correlates of survival and treatment response have not been widely explored in patients with and without HIV infection. This study recruited women with cervical cancer undergoing definitive chemoradiation (CRT) in Botswana. Clinical characteristics and blood samples were collected. Flow cytometry was performed on samples prior to initiation, at completion, and 3 months after CRT. Logistic regression analysis identified immune markers that differed by HIV status and correlated with overall survival (OS). The study enrolled 131 consecutive women (HIV+ *N* = 89 and HIV− *N* = 42). From initiation to 3 months after CRT, a significant decrease in CD4 frequency (72%–60.55%, *P* < 0.001) and an increase in CD8 frequency (20.9%–31.5%, *P* < 0.001) were seen in women without HIV, whereas no significant changes in CD4 frequency (52.5%–50.9%) or CD8 frequency (39.9%–41.4%) were observed in those with HIV. Peripheral T cells underwent similar activation across the cohort regardless of HIV status. Improved OS was associated with reduced frequency of IL-2–expressing CD4 T-cell subsets. In women living with HIV, enhanced OS was associated with the presence of proinflammatory CD8 T cells. CRT induces peripheral T-cell activation and distinct cytokine profiles that differ by HIV status. Despite similar OS, HIV infection may differentially affect immune response to CRT in women with well-managed HIV.

**Significance:**

Chemoradiation affects the immune system of patients with cervical cancer with well-controlled HIV infection differently than those without HIV, yet their survival does not differ. This finding is an important step in understanding how management of HIV infection can modify cancer outcomes, particularly in settings with a high burden of HIV.

## Introduction

Cervical cancer is the fourth most common cancer in women worldwide and remains the leading cause of cancer-related death in women in low- and middle-income countries (LMIC; ref. 1). Women living with human immunodeficiency virus (HIV; WLWH) are more likely to harbor persistent human papillomavirus (HPV) infection with high-risk HPV serotypes (i.e., HPV 16 and 18) and subsequently are at higher risk of developing cervical cancer than their counterparts without HIV infection (2–4). The majority of WLWH from LMICs who develop cervical cancer present with locally advanced disease (5–7). Standard treatment of locally advanced cervical cancer consists of chemoradiation (CRT) with external beam radiation (RT) and concurrent weekly cisplatin-based chemotherapy followed by brachytherapy (8). The advent and increased accessibility of antiretroviral therapy (ART) has extended the lifespan of people living with HIV; however, women remain at an increased risk of developing cervical cancer even when HIV infection is well-controlled on ART (9, 10). Understanding the ways in which HIV infection influences cancer biology is critical to improving outcomes for women with cervical cancer worldwide.

Botswana is an LMIC in sub-Saharan Africa with a high rate of HIV infection, surpassing 23% of the population of women of reproductive age (11). Cervical cancer rates far outpace those of any other cancer affecting women or men (11, 12). Despite literature from high-income countries suggesting that HIV infection negatively affects treatment outcomes, patients in Botswana with well-managed HIV infection who are treated with curative intent have clinical outcomes comparable with those without HIV infection (7, 10, 13). However, WLWH represent two-thirds of all patients presenting with invasive cervical cancer, despite excellent adherence to ART (7). This contrasts with other HIV-associated malignancies, which demonstrated significant decline in incidence after widespread adoption of ART (14, 15). Additionally, WLWH present with cervical cancer at a significantly younger age than those without HIV (7). These discrepancies in WLWH compared with their counterparts without HIV infection suggest that viral presence continues to promote an immunologic environment supportive of carcinogenesis even in the setting of viral load suppression and normal CD4 counts.

Although there is a growing body of literature describing changes in the immunologic profiles of patients receiving treatment for cervical cancer, most are limited to broad characterizations of lymphocyte fluctuations and lack detail about behavior of specific subsets of CD4 and CD8 cells. Current evidence suggests that pretreatment lymphopenia, rapid lymphocyte decline from baseline during treatment, and poor lymphocyte recovery after treatment completion may be associated with worse prognosis (16). Despite cervical cancer status as an acquired immunodeficiency syndrome (AIDS)-defining illness, there are few reports that describe immune profiles of WLWH undergoing treatment for cervical cancer and virtually none that compare with that of patients without HIV, limiting the understanding of any differential impact of HIV infection on treatment-related immune responses. A single study from Thailand suggested that WLWH on ART experienced less immunosuppression compared with those not taking ART, whereas another study from the United States suggested that in patients with cervical intraepithelial neoplasia, T-cell exhaustion is observed more frequently in those with coinfection of high-risk HPV subtypes compared with those with low-risk HPV subtypes (17, 18). However, these studies are limited by their lack of a comparator group without HIV infection. There is a significant need for ongoing biomedical research in this field, particularly research carried out in LMICs where the burden of HIV infection is heaviest.

Expanding the understanding of immunologic changes these patients undergo during and after treatment may not only serve a prognostic value but also provide predictive insights into those at high risk of treatment failure who may benefit from intensified primary treatment or follow-up strategies. Despite research attempting to identify molecular, genetic, and immunologic targets, there are no commercially available predictive biomarkers for patients with locally advanced cervical cancer (19). Yet identification of predictors of treatment response or recurrence from peripheral blood could provide valuable clinical information from a sample easily collected in a clinic, an approach particularly appealing in regions with limited access to diagnostic technology that have a higher burden of HIV infection.

This study analyzed peripheral blood mononuclear cell (PBMC) samples from a cohort of patients with cervical cancer from Botswana with and without HIV infection who received CRT with curative intent. Comparison of major peripheral T-cell subsets and functional markers allowed for identification of correlates of improved survival. The findings of this work provide novel insights into the immune landscape of women with well-controlled HIV infection.

## Materials and Methods

### Patient enrollment and clinical data

Study was approved by the Institutional Review Board (IRB) of the University of Botswana, University of Pennsylvania IRB (IRB# 822683), and the Ministry of Health of Botswana and conducted in accordance with recognized ethical guidelines. Women >18 years of age with locally advanced cervical cancer treated with curative intent CRT were enrolled at Princess Marina Hospital and Gaborone Private Hospital under the “Ipabalele” study protocol after obtaining written informed consent (20). Patient data and blood samples were collected as previously described (20). Clinical data collected included age, HIV status, stage using 2009 International Federation of Gynecology and Obstetrics guidelines (ref. 21), total radiation dose defined as equivalent total dose in 2-Gy fractions (EQD2; ref. 22), total number of chemotherapy cycles completed, and vital status.

### Patient PBMC analysis

PBMC samples analyzed in this study came from a larger, clinically well-characterized cohort (20). For each individual patient, at least two longitudinal samples were analyzed, including an initial time point before the start of treatment (Initial), at least one posttreatment time point, end of treatment (EOT), and/or 3 months after EOT (M3). For this study, patient samples that had at least an M3 time point were used in order to control for post-CRT hematologic toxicity often observed immediately after treatment. Control samples were procured from the Human Immunology Core at the University of Pennsylvania and run in parallel to study samples in accordance with IRB approval from the University of Pennsylvania (IRB# 705906).

### PBMC handling

Cryopreserved human PBMCs were transferred from liquid nitrogen storage onto dry ice for transport and processing in a biosafety hood. Samples were thawed quickly in a 37°C water bath. Before completely thawing, the samples were transferred to 15-mL conical tubes containing RPMI medium at room temperature to dilute (1:10) DMSO from cryopreservation solution in the sample. Cells were pelleted by centrifugation at 400 × *g* for 7 minutes to remove excess cryopreservation mixture in media. Samples were treated with DNase (100 μg/mL) for ∼5 minutes, upon which cells were diluted again in media to remove excess DNase. Cells in single-cell suspension were resuspended in fresh culture media [RPMI medium containing HEPES and glutamine supplemented with 10% FBS and antibiotics penicillin (100 IU/mL)/streptomycin (100 µg/mL)]. Cells were counted, and viability was assessed by trypan blue staining under manual light microscopy. Cells were plated at concentrations between 0.5 and 1.5 × 10^6^ cells/well in 96-well U-bottom plates and rested overnight at 37°C, 5% CO_2_ incubating conditions before use. Samples were plated in duplicate for surface and intracellular cytokine staining (ICS).

### Flow cytometry

Rested PBMCs designated for ICS were incubated with the CD107 antibody and simultaneously stimulated in culture media with phorbol 12-myristate 13-acetate (Sigma-Aldrich) + ionomycin (Sigma-Aldrich) in the presence of Monensin (BD GolgiStop) for 5 hours at 37°C, 5% CO_2_. After incubation, all cells were washed in PBS before incubating in Live/Dead Aqua (Invitrogen) staining per the manufacturer’s protocol for 25 minutes on ice. Samples were subsequently surface stained in designated flow panels (see below) in 50 μL of wash buffer + antibody cocktail for 30 minutes at room temperature. Samples designated for ICS were fixed and permeabilized using the FoxP3 Transcription Factor Staining Buffer Kit (Thermo Fisher Scientific) according to the manufacturer’s protocols. Samples were stained with an ICS panel antibody cocktail (see below) diluted in Perm/Wash solution for 50 minutes on ice. Samples were washed and centrifuged 3 times prior to resuspension in wash buffer before flow cytometric analysis on a BD LSR II. Panels analyzed were as follows: *T-cell subtyping panel* used a cocktail consisting of the following antibodies: CD3 (UCHT1, Beckman Coulter), CD4 (13B8.2, Beckman Coulter), CD8 (SK1, BioLegend), CD56 (N901, Beckman Coulter), CD45RA (2H4, Beckman Coulter), CCR7 (150503, BD Biosciences), CD27 (1A4CD27, Beckman Coulter), CD28 (CD28.2, Beckman Coulter), and CD57 (NK-1, BD Biosciences). *Functional profiling panel* involved an ICS panel that involved surface staining with CD3 (UCHT1, Beckman Coulter), CD4 (13B8.2, Beckman Coulter), CD8 (SK1, BioLegend), CD56 (N901, Beckman Coulter), and CD57 (NK-1, BD Biosciences) and subsequent intracellular staining with CD107 (H4A3, BioLegend), IFNγ (B27, BD Biosciences), TNFα (Mab11, BD Biosciences), and IL-2 (MQ1-17H12, Invitrogen).

### Flow analysis

BD LSR II used to acquire samples is part of the Penn Cytomics & Cell Sorting Shared Resource Laboratory. Samples were analyzed on LSR II using standardized voltages for the designated panels used. A gating strategy example is shown in Supplementary Fig. S1. Compensation for antibodies and LIVE/DEAD staining was set up using UltraComp eBeads (Invitrogen) and ArC reactive beads (Invitrogen), respectively. Compensation was applied on the machine and was confirmed/modified after processing on FlowJo software as needed. FlowJo v9.9.6 (BD Biosciences) was used for all flow cytometry analysis. Raw data generated from FlowJo analysis was exported for subsequent biostatistical analysis.

### Statistical analysis

Analyses and figure creation were performed in R (v4.1.0) using raw flow cytometric data acquired from experiments as described in the “Flow analysis” section and exported from FlowJo. Overall survival (OS) analyses were conducted in R using the packages “survival” and “survminer” applying the Cox proportional hazard regression model for the whole cohort and for the subsets of patients stratified by the time points and/or the HIV status. Adjusted *P* values were derived using Bonferroni correction. OS was estimated using Cox proportional hazard regression and Kaplan–Meier methods (23). OS was stratified by high or low expression of immune markers to identify correlates associated with improved OS. Univariate and multivariate linear regression analyses were performed across patient treatment time points. For multivariate regression analysis, the Initial time point were analyzed by including age and stage as independent variables. For EOT and M3 time points, CRT data (radiation dose and chemotherapy cycles) were also included with age and stage as independent variables. A two-sample two-tailed *t* test was performed for each marker comparing patients by HIV status at each time point.

### Data availability

All data generated in this study are available upon request from the corresponding author.

## Results

### Clinical characteristics and outcomes of the study cohort

Between 2016 and 2020, 296 patients with histology-confirmed cervical cancer were enrolled, and 131 had samples available for analysis (Table 1; ref. 20). WLWH comprised 67.9% (*N* = 89) of the study cohort, and all had well-controlled infection on ART. The median baseline CD4 count for WLWH at initial visit was 454 cells/μL (IQR = 275–590), and most patients presented with an undetectable viral load (*N* = 67, 75.3%). Most patients presented with stage II or III disease, and the majority of patients received four or more cycles of chemotherapy and >80 Gy EQD2 of RT dose. At EOT, the median CD4 count had declined to 92 cells/μL (IQR = 75–136) and recovered to 173 cells/μL (IQR = 135–239) by M3.

**Table 1:** Clinical characteristics of study participants with locally advanced cervical cancer

HIV status	Positive, *N* = 89 (67.9%)	Negative, *N* = 42 (32.1%)
Age (years)		
<40	*N* = 23 (25.8%)	*N* = 4 (9.5%)
40–50	*N* = 46 (51.7%)	*N* = 13 (30.9%)
>50	*N* = 20 (23.5%)	*N* = 25 (59.5%)
Median age	44.0	53.5
Histology		
Squamous cell carcinoma	*N* = 77 (86.5%)	*N* = 38 (90.4%)
Adenocarcinoma	*N* = 7 (7.9%)	*N* = 2 (4.8%)
Other	*N* = 5 (5.4%)	*N* = 2 (4.8%)
FIGO stage		
I	*N* = 19 (20.2%)	*N* = 8 (19%)
II	*N* = 43 (48.3%)	*N* = 26 (61.9%)
III	*N* = 26 (29.2%)	*N* = 8 (19%)
IV	*N* = 1 (1.1%)	*N* = 0 (0%)
EQD2 (Gy)		
<70	*N* = 12 (13.5%)	*N* = 6 (14.3%)
70–80	*N* = 12 (13.5%)	*N* = 3 (7.1%)
>80	*N* = 65 (73%)	*N* = 33 (78.6%)
Cycles of chemotherapy		
1	*N* = 1 (1.1%)	*N* = 1 (2.4%)
2	*N* = 5 (5.4%)	*N* = 2 (4.8%)
3	*N* = 18 (20.2%)	*N* = 5 (11.9%)
4	*N* = 35 (39.3%)	*N* = 10 (23.8%)
5	*N* = 30 (33.7%)	*N* = 24 (57.1%)
Vital status		
Alive	*N* = 60 (67.4%)	*N* = 27 (64.3%)
Deceased	*N* = 29 (32.6%)	*N* = 15 (35.7%)
Recurrence	*N* = 3 (3.4%)	*N* = 5 (11.9%)
Median CD4 (cells/μL), IQR		
Initial	454, 275–590 (*N* = 88)	928, 653–1,007 (*N* = 3)
EOT	92, 75–136 (*N* = 65)	N/A
M3	173, 135–239 (*N* = 63)	165, 152–178 (*N* = 2)
Viral load at initial visit		
Detectable	*N* = 22 (24.7%)	
Undetectable	*N* = 67 (75.3%)	
PBMC samples by time point		
Initial	*N* = 89	*N* = 42
EOT	*N* = 66	*N* = 33
M3	*N* = 64	*N* = 39

Abbreviations: EQD2, equivalent dose in 2-Gy fractions; FIGO, International Federation of Gynecology and Obstetrics.

The median follow-up was 36.8 months (IQR = 20.43–47.77). At the last follow up, 22% (*N* = 29) were deceased. Two-year OS was 77.9% [95% confidence interval (CI), 70%–84%] for the overall cohort, 78.4% (95% CI, 69%–85%) for WLWH, and 77.0% (95% CI, 63%–86%) for those without HIV (*P* = 0.865; Supplementary Fig. S1). There was no survival difference between patients with and without HIV infection (Supplementary Fig. S2).

### Frequency of peripheral CD4 and CD8 T cells in patients with cervical cancer during treatment

**Figure 1:**
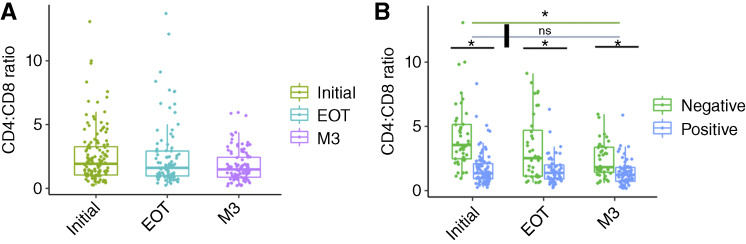
Changes in the CD4:CD8 ratio before and after CRT. Frequency of CD4 and CD8 T-cell subsets by flow cytometry. Box plots overlay individual patient data points for CD4 frequency divided by CD8 frequency (CD4:CD8 ratio) over three longitudinal study time points (Initial, EOT, and M3) in the (**A**) total cohort and (**B**) cohort stratified by HIV status. Significance (*P* < 0.05) indicated by *; blue and green lines indicate statistical comparison between Initial and M3 time points for the cohorts with and without HIV, respectively. ns, not significant.

Peripheral T-cell repertoires of the overall cohort displayed a narrowing of the peripheral CD4:CD8 T-cell ratio from Initial to EOT [1.9:1 (IQR = 1.1–3.3) to 1.6:1 (IQR = 1.0–2.9)], which was sustained at M3 (1.5:1, IQR = 0.9–2.4; Fig. 1A; Supplementary Table S1a). At Initial, WLWH presented with a narrower CD4:CD8 ratio (1.3:1, IQR = 0.9–2.1) compared with patients without HIV (3.4:1, IQR = 2.5–5.1; Fig. 1B; Supplementary Table S1b). At EOT, CD4 frequency significantly decreased and CD8 frequency significantly increased in women without HIV (Fig. 1B), resulting in a narrower CD4:CD8 ratio (2.5:1, IQR = 1.1–4.7), but the CD4:CD8 ratio in WLWH was unchanged (1.4:1, IQR = 0.9–2.0). A further narrowing was observed in women without HIV at M3 (1.9:1, IQR = 1.4–3.4); however, no significant change in the CD4 frequency, CD8 frequency, or CD4:CD8 ratio from baseline to M3 was observed in WLWH (1.2:1, IQR = 0.7–1.8). Univariate and multivariate regression analysis confirmed that the fold change in the CD4 frequency and CD4:CD8 ratio from Initial to M3 was significantly larger in patients without HIV compared with WLWH (*P* < 0.001; Supplementary Table S2).

### Activation of peripheral CD4 and CD8 T cells in patients with cervical cancer

**Figure 2:**
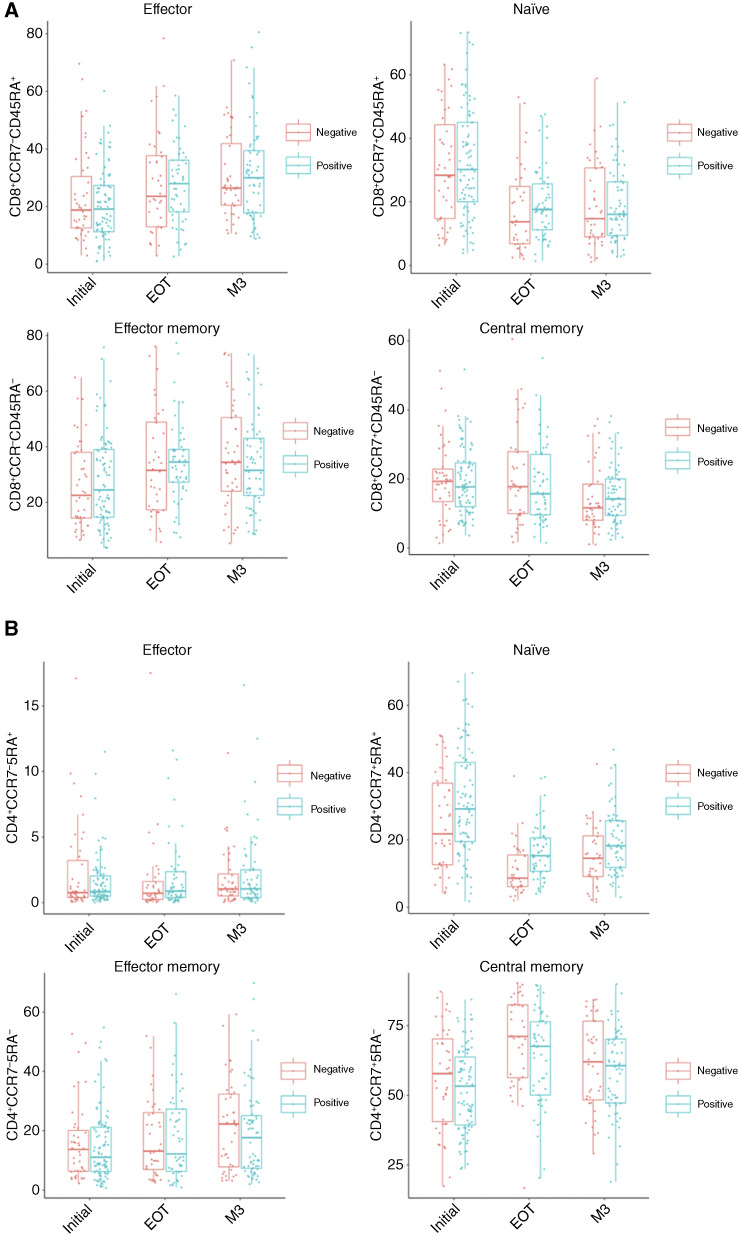
Changes in T-cell subsets before and after CRT by HIV status. **A,** CD8 T cells and (**B**) CD4 T cells were stratified into four major subsets by two additional markers (CCR7 and CD45RA) to identify naïve (CD45RA^+^CCR7^+^), central memory (CD45RA^−^CCR7^+^), effector memory (CD45RA^−^CCR7^−^), and effector (CD45RA^+^CCR7^−^) subsets. Box plots overlay individual patient data points for subset frequencies over three longitudinal study time points (Initial, EOT, and M3).

Expression of CCR7 and CD45RA on T cells were followed during treatment to track changes of four primary subtypes (naïve: CCR7^+^CD45RA^+^; central memory: CCR7^+^CD45RA^−^; effector memory: CCR7^−^CD45RA^−^; and effector: CCR7^−^CD45RA^+^; Fig. 2A and B). Activation markers associated with terminal differentiation (CD57) and cell proliferative capacity and memory phenotypes (CD27 and CD28) were measured given their prognostic value in literature characterizing tumor-infiltrating lymphocytes (TIL; refs. 24, 25). Activation of T cells were observed across all patients during treatment with loss in naïve subsets and increases in effector (CCR7^−^) subsets, although this was less pronounced in WLWH (Fig. 2A and 2B; Supplementary Fig. S3). By EOT, peripheral CD8 T cells showed statistically significant changes toward more activated phenotypes, characterized by an increase in populations that have lost CCR7 expression (i.e., effector and effector memory subsets) and reduction in the naïve (CCR7^+^CD45RA^+^) pool (Supplementary Table S1a; Supplementary Fig. S3). Additionally, markers of activation such as CD57 also increased during CRT (Supplementary Tables S1a and S2). Fold differences in frequencies of T-cell subsets suggest that WLWH present with more proinflammatory CD8 and CD4 T-cell activation signatures (increases in CD27^+^ and/or CD57^+^) on subsets that remain elevated and significantly different through M3 (Supplementary Table S1b). Although a higher frequency of peripheral proinflammatory T-cell subsets was observed in WLWH before and throughout CRT, overall few of these subsets are statistically different by HIV status.

### Cytokine expression by total CD4 and CD8 T cells

Differences in cytokine expression were assessed to assess global T-cell proinflammatory capacity. Across the entire cohort, total cytokine–expressing CD8 (IFNγ and TNFα, *P* < 0.001) and CD4 T (IFNγ, TNFα, and IL-2, *P* < 0.01) cells undergo similar expression kinetics from Initial to M3 (Supplementary Table S3a; Supplementary Fig. S4A and S4B). Frequencies of IFNγ- and TNFα-coexpressing CD8 T cells and IFNγ-coexpressing, IL-2–coexpressing, and TNFα-coexpressing CD4 T cells increase during CRT. At EOT, T cells from WLWH had more subsets expressing proinflammatory markers like IFNγ and TNFα by univariate and multivariate regression analyses and undergo significant increases in IFNγ- and TNFα-expressing CD4 and CD8 T-cell subsets from initial to M3 (Supplementary Table S3b).

**Figure 3:**
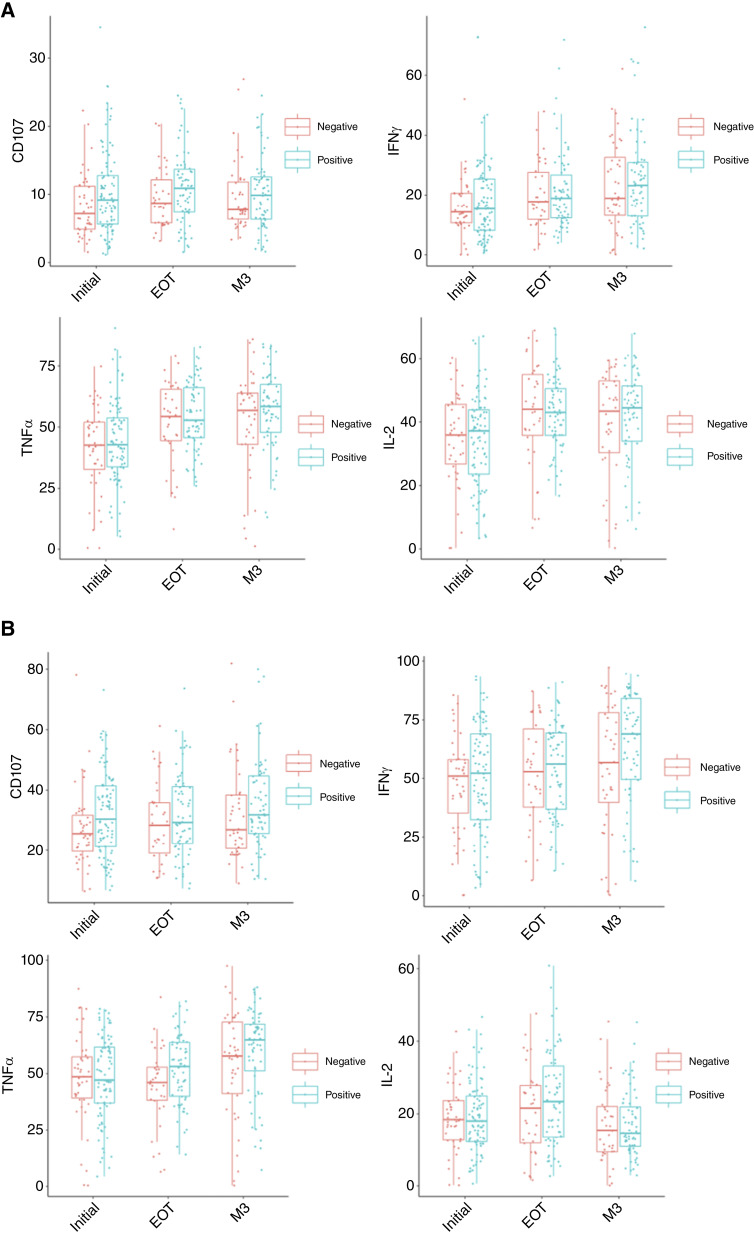
Changes in T-cell cytokine expression before and after CRT by HIV status. **A,** CD8 T cells and (**B**) CD4 T cells were stained for four primary effector/cytokine markers (CD107a, IFNγ, TNFα, and IL-2). Box plots overlay individual patient data points for total cytokine–expressing frequencies for T-cell subsets over three longitudinal study time points (Initial, EOT, and M3).

Multivariate regression analysis including age, HIV status, International Federation of Gynecology and Obstetrics stage, and treatment cycles showed WLWH exhibited more cytotoxic cells at initial visit and maintained a higher frequency of CD4 and CD8 T cells capable of expressing cytokines at M3 compared with those without HIV (Supplementary Table S2). It also showed higher total IL-2–expressing (HIV+: 44.45, IQR = 33.98–51.43 and HIV−: 43.5, IQR = 30.35–53) and TNFα-expressing (HIV+: 58.4, IQR = 47.85–67.45 and HIV−: 56.9, IQR = 42.98–63.88) CD4 T cells, and higher total IFNγ (HIV+: 69, IQR = 49.6–84.1 and HIV−: 56.7, IQR = 39.78–78.1) and TNFα (HIV+: 64.95, IQR = 51.25–71.83 and HIV−: 57.8, IQR = 41.15–72.78) expression by CD8 T cells (Fig. 3A and B; Supplementary Table S4). In WLWH, these changes included CD4 T cells with predominant populations expressing IL-2 as well as CD8 T cells with significant increases in proinflammatory markers such as IFNγ and TNFα (Supplementary Table S3b). Overall, CD8 T cells from WLWH maintained a proinflammatory status by EOT. CD4 and CD8 T-cell polyfunctionality increased during CRT regardless of HIV status (Supplementary Table S2). Taken together, WLWH present with a larger proinflammatory repertoire before and during CRT, with CD4 polyfunctionality increasing by M3 regardless of HIV status.

### T-cell markers associated with OS

**Figure 4:**
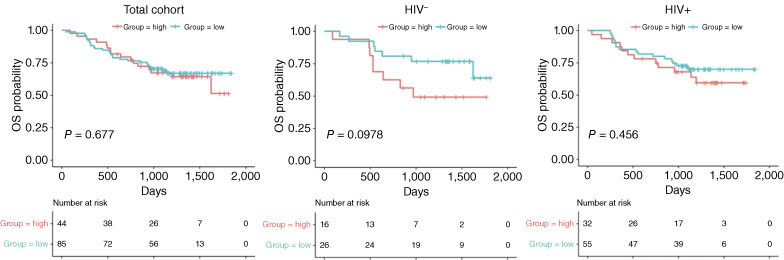
Correlation of the CD4:CD8 ratio with patient OS. Kaplan–Meier curves show OS probabilities by peripheral CD4:CD8 ratio for the total analyzed cohort and by HIV status. Ratios are designated as those lower than the mean (blue) vs. higher than the mean (red).

At initial visit, subsets associated with increased OS in the overall cohort included higher frequency in two CD27^+^CD8^+^ T-cell subsets and lower frequency of two CD8 subsets with activated phenotypes (Supplementary Table S2, Initial, Total). Patients without HIV that presented initially with lower frequency of activated CD4 T-cell subsets and activated CD8 T cells had better OS. Changes in peripheral CD4:CD8 ratios in women without HIV was more striking during CRT compared with WLWH, but this did not statistically correlate with OS (Fig. 4). Improved survival in WLWH was associated with activated subsets at Initial, including CD4^+^ T cells expressing IFNγ and IL-2 at higher frequency as well as lower frequency of non-differentiated CD8 subtypes like CD57-naïve cells (Supplementary Table S2). Women without HIV experienced better outcomes with CD4^+^ T-cell subsets with lower cytokine expression profiles (Supplementary Table S4). In the total cohort, improved OS was associated with activated CD8 subsets lacking CD27 and CD28 at EOT. When stratified by HIV status, patients without HIV who had lower frequency of IL-2–expressing CD4 T-cell subsets had better OS. These subsets were present at a higher frequency in WLWH (Supplementary Table S2). Many T-cell subsets expressing IL-2 significantly decreased between initial and M3 (Supplementary Table S3a). Lower frequency of multiple IL-2–expressing T-cell subsets was associated with better OS at all three time points regardless of HIV status (Supplementary Table S5).

## Discussion

The aim of this study was to investigate differences in the immunologic response to CRT by a cohort of patients with cervical cancer with a large proportion of WLWH with well-managed HIV infection. Analysis of peripheral T-cell repertoires before and after treatment demonstrated distinct differences in the immune profiles of WLWH compared with their counterparts without HIV infection. This included a narrower initial CD4:CD8 ratio that remained relatively stable over treatment compared with the significant decrease seen in patients without HIV, increased T-cell activation at baseline and throughout treatment compared with patients without HIV, and disparate predictors of survival despite similar 2-year OS, AA with immune activation being protective in WLWH but detrimental in those without HIV. These results suggest that WLWH who are treated with CRT have an immunologic profile distinct from their counterparts without HIV, yet these differences are not substantial enough to ultimately affect OS after treatment.

A narrow CD4:CD8 ratio close to 1 has been suggested to represent high levels of immune activation, T-cell exhaustion, and T-cell replicative senescence in patients living with HIV (26). People living with HIV who have a narrower CD4:CD8 ratio are at higher risk of developing cancer. WLWH who present with cervical cancer in Botswana often have CD4 counts well above AIDS-defining levels (i.e., >200 cells/mm^3^); however, they typically remain below that of their counterparts without HIV (27, 28). The study found that the CD4 count as measured by routine HIV monitoring laboratory tests in the WLWH cohort demonstrated fivefold reduction in CD4 at EOT and recovery to just under half of baseline at 3 months. WLWH had a narrower CD4:CD8 ratio at all time points compared with those without HIV, but that CD4:CD8 ratio kinetics differed between the two groups. Patients without HIV had a significant decrease in CD4:CD8 ratio and associated reduction in CD4 T-cell frequency and increase in CD8 T-cell frequency during CRT, whereas the CD4:CD8 ratio as well as CD4 and CD8 T-cell frequencies remained stable in WLWH. Prior studies on the effect of CRT on peripheral lymphocytes are consistent with this finding in patients without HIV, demonstrating that women receiving treatment typically undergo a significant decrease in peripheral CD4 frequency and CD4:CD8 ratio, resulting in prolonged clinical lymphocytopenia (29–33). However, the lack of variation in the CD4:CD8 ratio and CD4 and CD8 T-cell frequency observed here in WLWH has not previously been reported.

Despite these differing patterns of CD4 and CD8 cell frequencies in WLWH, T-cell subsets still underwent increased activation in patients regardless of HIV status, although was more pronounced in WLWH. CD4 and CD8 T cells trended toward loss of naïve populations and had a corresponding shift toward activated effector populations during CRT that continued to M3. This corresponded with an increase in T cells expressing inflammatory cytokines, which was similarly more pronounced in WLWH.

These findings prompt several theories about the differences observed between WLWH and those without HIV. Peripheral T cells may experience heightened activation prior to treatment initiation, an observation that would be consistent with an environment of chronic immune activation and CD4 T-cell depletion that is associated with HIV infection (34). It may also be that WLWH lack a distinct subpopulation of T cells that has increased sensitivity to CRT compared with those without HIV. Further investigation with extensive lymphocyte subtyping, for example including regulatory T-cell populations and antigen-specific populations, would be needed to characterize this phenomenon. Overall, the immunologic distribution patterns observed in WLWH are likely due to multifactorial causes that may include loss of broad or specific CD4 T-cell subsets, increase in CD8 T cells during treatment response, or a combination of both mechanisms. HIV directly affects CD4 T cells and indirectly impairs CD8 T-cell functionality due to dependence on CD4 interaction for activation. Activated T cells undergo drastic shifts in metabolism and division and are likely differentially sensitive to these treatments. Understanding the sensitivity of T-cell subsets to CRT within our patient cohort could provide insights into the mechanisms that reshape immune repertoires regardless in patients both with and without HIV.

OS in this cohort was consistent with prior reports in this population and did not differ by HIV status (10). Survival of the overall cohort was positively associated with a higher frequency of activated CD8 subsets at baseline, a higher frequency of activated CD8 subsets at EOT, and a lower frequency of IL-2–expressing CD4 or CD8 subsets at M3. Within the cohort of WLWH, a higher frequency of activated CD8 cells and CD4 subsets expressing IFNγ and IL-2 at baseline and lower frequency of IL-2–expressing cells at M3 was associated with improved survival. Taken together, these results suggest that survival is positively associated with an activated pretreatment and tempered posttreatment immune profile. A pattern of persistent activation can be consistent with the chronic inflammation related to prolonged HIV infection. Although inflammatory cytokine profiles positively correlate with the viral load, this trend is not completely abrogated by appropriate management with ART, resulting in a low but persistent inflammatory state even in people with no detectable viral load (34, 35). Chronic inflammation in patients living with HIV has been associated with T-cell exhaustion and higher susceptibility to cancer development and therefore may also play a role in treatment outcomes (34). Although not currently in regular clinical use, IL-2 has historically been trialed as a cancer therapeutic as well as for use in immune fortification for patients with HIV infection (36–39). Although IL-2 has pleiotropic effects on immune proliferation and homeostasis, its role in the posttreatment immunologic landscape for the patients in this study are not clear. However, reduced levels at M3 may indicate a pattern of inflammatory decline that is protective of entering a chronic inflammatory state. Further studies will be needed to characterize the optimal immune environment for patients before, during, and after treatment in order to determine if immune system modulation can affect disease outcomes.

Little is known about whether CRT differentially affects specific T-cell populations at the site of disease versus the periphery. Transient depletion of CD4 T cells has been described in novel anticancer therapies to remodel the immune repertoire (40). CRT-mediated immune repertoire remodeling may be one mechanism by which well-managed WLWH respond to treatment. CRT, particularly focal radiotherapy, can convert “cold” tumor cells into “*in situ*, individualized vaccines” for priming tumor specific CD8 T cells (41, 42). Activated tumor-infiltrating immune cell populations (43) have been reported as potential immune correlates for improved patient outcomes (24, 44). The restoration of functional CD4 populations by ART coupled with increased CRT-mediated tumor killing may provide a synergistic role in WLWH. This study sets the stage for future studies interrogating antigen-specific differences both in the vascular periphery and tumor microenvironment to determine if these correlates have utility in assessing tumor-specific responses and patient outcomes.

Although this study provides important novel findings about the immune profile of patients with cervical cancer undergoing CRT, there are several limitations. Given the well-controlled nature of HIV infection in this cohort, patterns of immune expression reported here are not generalizable to patients with newly diagnosed or poorly controlled HIV infection. Due to limitations in sample size, analysis of the patterns of regulatory T cells during CRT was not possible. Studies examining comparisons of the peripheral immune kinetics with those of TILs in the cervical microenvironment were not done because of IRB limitations. However, these limitations represent a rich area of study for the future. Other areas of future focus may include analysis with HPV or HIV antigen-specific T-cell identification to better understand the kinetics of peripheral lymphocytes with tumor-specific functions that may have more closely reflected the role of TILs as well as investigation of the impact of CRT on immune subsets associated with exhaustion and regulatory function.

In conclusion, this study demonstrates that standard CRT induces a significant pattern of activation and differentiation in T cells present in peripheral blood of patients with cervical cancer during treatment regardless of HIV status. However, lymphocyte kinetics differ between patients with and without HIV infection, and WLWH have a higher activation profile at baseline. Although WLWH may be at higher risk of T-cell exhaustion after treatment, they do not experience inferior survival outcomes in this cohort with well-managed HIV infection. Understanding the impact of CRT on immune response is important in all patients with cervical cancer and particularly those with HIV infection who may have the opportunity to optimize HIV infection control prior to CRT initiation.

## Supplementary Material

Supplementary Tables 1-5 Supplementary Tables 1-5

Supplementary Figure 1 Supplementary Figure 1

Supplementary Figure 2 Supplementary Figure 2

Supplementary Figure 3 Supplementary Figure 3

Supplementary Figure 4 Supplementary Figure 4
