# Supplementary material for: Patients with Cervical Cancer with and without HIV Infection Have Unique T-cell Activation Profiles despite Similar Survival Outcomes after Chemoradiation
Source: Cancer Res Commun. 2025 Apr 14;5(4):610–20. doi: 10.1158/2767-9764.CRC-24-0364 (PMC11995389; doi:10.1158/2767-9764.CRC-24-0364)
Supplement: Supplementary Tables 1-5 [file crc-24-0364_supplementary_tables_1-5_suppst1-st5.pdf]

Supplemental Tables

Table S1a. Frequency of major peripheral T cell subsets at three treatment timepoints for all patients. Abbreviations: EOT, End of treatment visit; M3, 3 months post treatment visit; IQR, Interquartile Range. P-values (pvals) derived using student t-test comparing difference in marker frequency from Initial to M3. Significance displayed as asterisks where ns= no significance, *=p<0.05, **=p<0.01, ***=p<0.001. Control patients are non-matched female normal donors from the UPenn Human Immunology core (HIC).

Patient Timepoint	Initial (N=131)		EOT (N=99)		M3 (N=113)		
	**Frequency (%)**	**IQR**	**Frequency (%)**	**IQR**	**Frequency (%)**	**IQR**	**Initial to M3 (pval)**
CD4:CD8 Ratio	1.92	1.05-3.25	1.61	0.96-2.92	1.49	0.86-2.44	***
CD8+ %	31.60	21.23-45.28	34.20	23.3-46.3	37.15	26.8-48.33	***
CD8+CCR7+ %	51.85	37.1-66.98	37.30	22.92-49	32.20	20.18-44.54	**
CD8+ CCR7- %	48.15	33-62.95	62.70	51-77.1	67.75	55.48-79.83	***
Naïve CD8 % (CCR7+CD45RA+)	29.90	18.38-45.08	17.20	8.29-25.7	15.80	9.3-27.08	***
Central Memory CD8 % (CCR7+CD45RA-)	18.50	12.35-24.25	16.60	9.74-27.6	12.70	8.72-19.9	***
Effector Memory CD8 % (CCR7-CD45RA-)	23.70	14.43-38.8	26.20	23.9-42.7	33.30	22.6-45.6	***
Effector CD8 % (CCR7-CD45RA+)	18.95	11.43-28.18	33.40	16.1-37.1	27.75	17.98-39.93	***
CD8+CD57+ %	29.45	20.73-42.6	40.50	30.02-53.71	51.40	38.08-62.71	***
CD8+CD28+CD27+ %	49.00	34.9-65.825	31.30	19.6-42.8	32.20	22.07-43.47	ns
CD8+CD28+CD27- %	6.32	4.17-10.075	6.37	3.91-9.94	9.22	5.4175-16.4	ns
CD8+CD28-CD27+ %	7.49	4.3625-11.85	12.00	7.72-16.5	7.15	4.24-11.70	ns
CD8+CD28-CD27- %	32.60	18.5-45.75	45.60	35.5-56.3	45.85	22.07-43.47	ns
CD4+ % %	60.50	48.1-70.75	54.70	43.8-68.1	54.70	42.25-65.45	**
CD4+CCR7+ %	86.20	76.59-92.55	86.36	70.3-91.86	79.60	68.85-91.63	*
CD4+ CCR7- %	13.86	7.42-23.4	13.62	8.157-29.73	20.44	8.37-31.19	*
Naïve CD4 % (CCR7+CD45RA+)	27.45	18.13-41.4	12.40	8.23-18.4	16.95	10.75-24.95	***
Central Memory CD4 % (CCR7+CD45RA-)	54.10	39.83-65.9	69.30	53.3-79.9	61.00	47.3-70.93	**
Effector Memory CD4 % (CCR7-CD45RA+)	12.05	6.37-20.88	12.80	6.28-27.2	19.40	7.42-28.73	**
Effector CD4 % (CCR7-CD45RA-)	0.83	0.4-2.16	0.80	0.347-2.13	1.04	0.37-2.48	ns
CD4+CD57+%	8.77	4.92-13.46	11.67	8.06-17.3	12.52	7.86-17.8	**

Table S1b. Frequency of major peripheral T cell subsets at three treatment timepoints by HIV status. Abbreviations: EOT, End of treatment visit; M3, 3 months post treatment visit; IQR, Interquartile Range; P-values (pvals) compare marker frequency between Initial and M3. Significance indicated as ns= no significance, *=p<0.05, **=p<0.01, ***=p<0.001.

HIV Status	Positive							Negative						
PBMC Timepoint	Initial (N=89)		EOT (N=66)		M3 (N=64)		Initial to M3 (pval)	Initial (N=42)		EOT (N=33)		M3 (N=39)		Initial to M3 (pval)
	**Frequency (%)**	**IQR**	**Frequency (%)**	**IQR**	**Frequency (%)**	**IQR**		**Frequency (%)**	**IQR**	**Frequency (%)**	**IQR**	**Frequency (%)**	**IQR**	
CD4:CD8 Ratio	1.32	0.94-2.1	1.36	0.89-2.01	1.23	0.72-1.8	ns	3.44	2.48-5.14	2.54	1.12-4.69	1.92	1.42-3.38	***
CD8+ %	39.90	29.3-47.9	37.70	28.4-47.3	41.45	32.43-53.025	ns	20.90	15.5-27.1	25.45	15.9-39.9	31.55	21.25-38.95	***
CD8+CCR7+ %	51.80	37.3-66.8	37.30	24.98-48.28	32.25	20.59-46.67	***	52.10	33.88-69.25	35.42	19.26-57.07	31.18	19.29-43.5	***
CD8+ CCR7- %	48.24	33.15-62.7	62.70	51.7-75.1	67.70	53.28-79.45	***	47.88	30.7-66.1	64.65	42.93-80.65	68.75	56.575-80.67	***
Naïve CD8 % (CCR7+CD45RA+)	30.20	20-45.05	17.60	11.2-25.7	18.25	9.4-26.35	***	28.40	14.8-44.3	13.70	6.815-24.85	14.75	8.9225-30.7	***
CM CD8 % (CCR7+CD45RA-)	17.70	11.95-24.65	15.70	9.65-27.1	14.25	9.435-20.05	*	19.30	13.4-22.85	17.75	9.935-27.925	11.65	8.03-18.53	*
EM CD8 % (CCR7-CD45RA-)	24.50	14.7-39.05	34.50	18.2-36.1	31.55	17.8-39.375	*	22.60	14.35-38	31.55	17.325-48.925	34.45	24.03-50.6	**
Effector CD8 % (CCR7-CD45RA+)	19.10	11.25-27.35	28.00	27.3-39.1	30.00	22.5-43.075	***	18.80	12.55-30.45	23.60	12.95-37.675	26.45	20.45-41.85	*
CD8+CD57+ %	30.84	23.61-46.02	41.11	31.68-54.79	51.88	40.15-63.74	***	27.56	20.19-40.35	38.54	27.38-45.83	50.48	35.28-59.25	***
CD8+CD28+CD27+ %	49.10	34.75-65.25	31.30	23.1-39.8	30.70	22.07-41.03	ns	48.00	35.7-69	29.90	18.05-49.13	33.60	22.15-46.4	ns
CD8+CD28+CD27- %	6.71	4.44-10.15	6.03	3.91-8.99	9.08	5.35-16.1	ns	5.52	3.49-9.79	6.89	4.24-11.65	10.25	5.6-16.43	ns
CD8+CD28-CD27+ %	7.29	4.44-11.4	12.70	9.53-17.3	7.49	4.81-12.425	ns	8.03	4.23-13.4	9.23	6.93-14.9	6.01	3.44-10.43	ns
CD8+CD28-CD27- %	31.30	19.45-46.7	44.80	37.4-54.7	45.00	37.1-57.13	ns	33.10	17.15-44	49.65	27.73-59.43	47.00	33.05-62.07	ns
CD4+ %	52.50	44.15-62.55	51.20	41.3-60.2	50.95	39.07-57.17	ns	72.00	66.75-79.35	64.70	49.43-77.33	60.55	53.93-72.33	***
CD4+CCR7+ %	86.85	76.33-92.55	86.90	70.2-91.86	81.89	69.51-91.55	ns	85.70	78.35-92.4	86.30	72.03-92.3	76.05	66.12-91.73	ns
CD4+ CCR7- %	13.13	7.47-23.69	13.13	8.157-29.79	18.13	8.38-30.59	ns	14.27	7.59-21.64	13.69	7.72-27.97	23.97	8.29-33.84	ns
Naïve CD4 % (CCR7+CD45RA+)	29.20	19.5-43.05	15.30	10.7-20.6	18.25	11.775-25.725	***	21.80	12.65-36.9	8.66	6.13-15.5	14.60	9.065-21.25	***
CM CD4 % (CCR7+CD45RA-)	53.30	39.35-63.75	67.60	50.1-76.4	60.60	47.275-70.2	**	57.80	40.55-70.25	71.10	56.35-82.47	62.00	48.35-76.63	ns
EM CD4 % (CCR7-CD45RA+)	11.10	6.27-21.2	12.20	6.28-27.3	17.65	0.37-2.5	ns	13.70	6.375-20.15	13.15	6.97-26.15	22.30	7.853-32.37	ns
Effector CD4 % (CCR7-CD45RA-)	0.83	0.48-2.03	0.87	0.378-2.36	1.04	7.42-25.1	ns	0.76	0.39-3.21	0.71	0.24-1.59	1.03	0.5-2.18	ns
CD4+CD57+ %	9.47	6.41-15.07	13.16	8.6-21.84	13.67	8.78-20.54	*	6.60	4.33-9.81	9.63	6.722-13.96	10.54	7.08-16.61	**

Table S2. Differential T cell marker expression by HIV status. A) Univariate and B) Multivariate regression analysis was performed on flow data for T cell subtyping markers. Fold (HIV+/HIV-) denotes the ratio of median marker frequency where expression is greater in HIV+ when fold is >1 and greater in HIV- when <1. Significance indicated as ns= no significance, *=p<0.05, **=p<0.01, ***=p<0.001.

Analysis	Timepoint	T cell subtype	Fold change (HIV+/HIV-)	pval
Univariate	Initial	CD4+ Freq	0.72916667	***
		CD8+ Freq	1.90909091	***
		CD4:CD8 Ratio	0.38316667	***
		CD4+CD57-CD27-	0.6754386	**
		CD4+CD57+CD27+	1.78021978	**
		Total CD4+CD57+	1.43528342	*
		CD4+CCR7+CD45RA+	1.33944954	*
		CD8+CD57- Total CD27+CD28-	1.62150538	**
		CD8+CCR7+ Total CD27+CD28-	1.84482759	*
		CD8+CCR7+ Total CD27-CD28+	0.66478076	*
		CD8+CD57+ Total CCR7-CD45RA+	0.67087156	*
	EOT	CD4+ Freq	0.79134467	**
		CD4+CCR7+CD45RA+	1.7677643	**
		CD8+ Freq	1.48133595	**
		CD4:CD8 Ratio	0.53421013	*
		CD8+CD57- Total CCR7+CD45RA-	0.80143113	*
	M3	CD4+ Freq	0.84145334	***
		CD4:CD8 Ratio	0.64047896	***
		CD8+ Freq	1.31378764	***
		CD4+CD57+CD27+	1.48689139	**
		CD4+CCR7+CD45RA+	1.25	*
		CD8+CD57- Total CD27+CD28-	1.35458167	*
		CD8+CCR7+ Total CD27+CD28-	1.70207254	*
Multivariate	Initial	CD8+ Freq	1.90909091	***
		CD4+ Freq	0.72916667	***
		CD4:CD8 Ratio	0.38316667	***
		CD4+CD57-CD27-	0.6754386	*
		Total CD4+CD57+	1.43528342	*
		CD4+CD57+CD27+	1.78021978	*
		CD8+CCR7+ Total CD27+CD28-	1.84482759	*
		CD8+CD57- Total CD27+CD28-	1.62150538	*
	EOT	CD8+ Freq	1.48133595	**
		CD4+ Freq	0.79134467	**
		CD4:CD8 Ratio	0.53421013	*
		CD4+CCR7+CD45RA+	1.7677643	*
		CD8+CD28-CD27+	1.375948	*
	M3	CD8+ Freq	1.31378764	***
		CD4+ Freq	0.84145334	***
		CD4:CD8 Ratio	0.64047896	***
		CD4+CD57+CD27+	1.48689139	***
		Total CD4+CD57+	1.29696395	**
		CD4+CD57-CD27-	0.85192698	*

Table S3a. Frequency of cytokine expressing T cell subsets at three treatment timepoints for all patients. Cytokine expressing T cell subsets described reflect cells only expressing the cytokines labeled unless denoted as “Total” expressors. Abbreviations: EOT, End of treatment visit; M3, 3 months post treatment visit; IQR, Interquartile Range; P-values (pvals) compare marker frequency between Initial and M3. Significance indicated as ns= no significance, *=p<0.05, **=p<0.01, ***=p<0.001.

Patient Timepoint	Initial (N=131)		EOT (N=99)		M3 (N=113)		Initial to M3 (pval)
	**Frequency (%)**	**IQR**	**Frequency (%)**	**IQR**	**Frequency (%)**	**IQR**	
No Cytokine CD8	34.81	20.95-38.13	28.64	21.33-40.13	22.81	23.75-42.83	**
CD8+ Total CD107	28.35	31.95-66.95	28.95	37.15-69.43	30.9	45.2-80.68	ns
CD8+ Total IFNg	52.05	12.3-24.55	55.3	12.68-30.53	66.05	10.22-21.98	***
CD8+ Total TNFa	48.35	37-61.05	50.1	39.2-61.4	61.9	44.93-72.28	***
CD8+ Total IL-2	18.2	22.27-46.5	22.8	19.56-38.82	14.9	12.79-35.99	ns
CD107+	2.97	1.86-5.07	2.5	1.4-4.31	1.95	1.06-3.32	**
IFNg+	2.59	1.61-6.09	5.7	3.51-10.19	3.24	1.4-8.17	*
IL2+	2.11	1.23-3.05	2.03	1.26-3.35	1.32	0.77-2.19	**
TNFa+	2.34	1.23-3.74	1.83	1.19-3.4	1.65	0.94-3.23	*
CD107+IFNg+	1.78	0.72-4.37	3.18	1.41-7.11	2.3	0.95-6.25	*
CD107+IL2+	0.11	0.04-0.25	0.14	0.03-0.28	0.07	0.02-0.15	***
CD107+TNFa+	0.47	0.26-0.8	0.42	0.19-0.7	0.38	0.19-0.86	ns
IFNg+IL2+	1.31	0.63-2.41	1.54	0.78-3.15	0.82	0.46-1.54	***
IFNg+TNFa+	14.52	9.2-20.92	14.76	8.73-19.41	20.91	15.15-30.51	***
IL2+TNFa+	1.99	1.27-3.54	3.11	1.6-5.67	1.99	1.14-4.4	ns
CD107+IFNg+IL2+	0.3	0.12-0.94	0.53	0.22-1.16	0.24	0.08-0.53	**
CD107+IFNg+TNFa+	12.36	6.05-18.89	11.02	6.36-16.26	17.05	9.87-23.52	**
CD107+IL2+TNFa+	0.19	0.08-0.42	0.35	0.17-0.83	0.24	0.09-0.48	ns
IFNg+IL2+TNFa+	6.31	3.62-8.51	6.47	3.66-9.6	5.07	3.18-8.54	ns
Quad	2.72	1.15-5.11	2.93	1.68-4.41	2.35	1.24-3.95	ns
No Cytokine CD4	42.89	5.46-12	33.07	6.44-13.4	32.05	6.33-12.3	***
CD4+ Total CD107	8.49	9.62-23.4	9.85	12.3-27.63	8.69	12.98-31.53	ns
CD4+ Total IFNg	15.35	25.33-44.5	18.15	35.65-52.28	21.3	33.18-52.2	***
CD4+ Total TNFa	42.8	33.6-52.8	53.35	44.9-66.18	58.05	46.05-66.7	***
CD4+ Total IL-2	36.5	33.99-54.78	43.3	25.28-45.18	44.25	25.34-44.02	**
CD107+	2.38	1.41-3.95	1.95	1.17-3.33	1.96	1.15-3.35	ns
IFNg+	0.44	0.25-0.96	0.8	0.4-1.98	0.54	0.26-1.03	ns
IL2+	7.59	3.1-12.82	5.62	3.43-10.11	4.94	2.32-8.3	***
TNFa+	8.1	5.42-11.78	9.36	6.26-12.04	8.4	5.6-11.38	ns
CD107+IFNg+	0.1	0.04-0.25	0.2	0.1-0.69	0.15	0.06-0.42	*
CD107+IL2+	0.13	0.05-0.29	0.16	0.06-0.31	0.12	0.04-0.21	*
CD107+TNFa+	0.51	0.29-0.81	0.53	0.24-0.82	0.51	0.31-0.82	ns
IFNg+IL2+	0.31	0.12-0.89	0.4	0.17-0.94	0.24	0.08-0.61	ns
IFNg+TNFa+	3.36	2.04-6.86	3.58	2.07-6.62	4.8	2.55-9	**
IL2+TNFa+	15.76	10.59-21.77	22.21	16.31-30.05	22.2	16.57-26.79	***
CD107+IFNg+IL2+	0.01	0-0.04	0.03	0-0.08	0.01	0-0.04	ns
CD107+IFNg+TNFa+	0.92	0.5-2.33	0.89	0.53-2.08	1.3	0.52-2.61	ns
CD107+IL2+TNFa+	0.46	0.2-0.91	0.92	0.35-1.61	0.71	0.26-1.32	*
IFNg+IL2+TNFa+	6.45	3.97-9.29	8.3	5.81-11.06	9.87	6.75-14.24	***
Quad	0.36	0.14-0.8	0.54	0.26-0.92	0.57	0.24-0.99	ns

Table S3b. Frequency of T cell cytokine expression at three treatment timepoints by HIV status.
Abbreviations: EOT, End of treatment visit; M3, 3 months post treatment visit; IQR, Interquartile Range) compare marker frequency between Initial and M3. Significance indicated as ns= no significance, *=p<0.05, **=p<0.01, ***=p<0.001.

HIV Status	Positive							Negative						
Patient Timepoint (N=Samples)	Initial (N=89)		EOT (N=66)		M3 (N=64)		**Initial to M3**	Initial (N=42)		EOT (N=42)		M3 (N=39)		**Initial to M3**
	**Frequency (%)**	**IQR**	**Frequency (%)**	**IQR**	**Frequency (%)**	**IQR**		**Frequency (%)**	**IQR**	**Frequency (%)**	**IQR**	**Frequency (%)**	**IQR**	
No Cytokine CD8	32.14	20.2-47.21	25.26	19.57-37.26	18.62	11.54-33.56	***	36.7	25.85-44.84	33.18	20.57-40.66	25.97	13.91-40.68	ns
CD8+ Total CD107	30.4	21.2-41.4	29.25	22.18-41.2	31.85	25.6-44.73	ns	25.5	19.65-31.65	28.35	19-35.85	26.85	20.55-38.43	ns
CD8+ Total IFNg	52.2	32.4-69	56.15	36.85-69.35	69	49.6-84.1	***	51	35.25-58.1	52.85	37.73-71.15	56.7	39.78-78.1	ns
CD8+ Total TNFa	47.2	37-61.7	53.1	40.08-63.75	64.95	51.25-71.83	***	48.7	39.25-57.45	46.05	38.23-52.85	57.8	41.15-72.78	ns
CD8+ Total IL-2	18	12.2-24.9	23.35	13.5-33.13	14.65	10.9-21.9	ns	18.4	12.7-23.65	21.5	11.85-27.8	15.4	9.43-21.98	ns
CD107+	3.08	1.88-5.8	2.24	1.34-3.82	1.95	1.06-3.48	ns	2.84	1.77-4.35	2.7	1.56-4.8	2.06	1.08-3.08	ns
IFNg+	2.75	1.76-6.24	6.33	3.36-10.19	3.96	1.92-7.68	*	1.98	1.49-4.66	4.83	3.66-9.82	2.25	1.19-8.18	ns
IL-2+	2.13	1.1-3.03	2.12	1.45-3.21	1.22	0.76-2.17	***	1.96	1.31-3.15	1.65	0.89-3.54	1.5	0.84-2.51	ns
TNFa+	2.04	1.14-3.59	1.83	1.21-3.28	1.75	0.92-3.07	ns	2.59	1.89-3.94	2.19	1.16-4.03	1.58	1.02-3.23	ns
CD107+IFNg+	2.39	1.03-4.85	3.31	1.58-6.99	2.81	1.55-6.48	ns	0.87	0.49-3.08	2.53	1.4-7.56	1.24	0.51-4.28	ns
CD107+IL-2+	0.14	0.05-0.28	0.16	0.04-0.25	0.07	0.02-0.12	***	0.08	0.03-0.13	0.1	0.02-0.32	0.07	0.01-0.15	ns
CD107+TNFa+	0.42	0.24-0.78	0.49	0.2-0.8	0.35	0.19-0.82	ns	0.51	0.31-0.88	0.4	0.19-0.64	0.43	0.18-0.98	ns
IFNg+IL-2+	1.21	0.63-2.53	1.66	0.87-3.18	0.86	0.52-1.46	***	1.4	0.65-2.14	1.15	0.78-2.81	0.78	0.43-1.8	ns
IFNg+TNFa+	13.75	9.07-20.86	14.09	8.63-19.35	21.49	15.74-32.29	***	16.73	10.19-21.07	16.32	8.95-19.38	20.14	12.74-28.17	ns
IL-2+TNFa+	1.93	1.25-3.26	2.93	1.65-5.76	1.92	1.14-3.93		2.6	1.31-4.43	3.59	1.22-4.96	2.06	1.13-4.46	ns
CD107+IFNg+IL-2+	0.37	0.12-1.12	0.53	0.23-1.18	0.26	0.13-0.52	***	0.22	0.11-0.54	0.52	0.18-1.13	0.18	0.08-0.55	ns
CD107+IFNg+TNFa+	12.65	5.61-19.01	11.63	6.59-17.98	17.18	10.15-24.47	**	11.91	7.78-17.51	9.37	5.3-13.63	15.5	8.74-20.08	ns
CD107+IL-2+TNFa+	0.19	0.1-0.44	0.36	0.19-0.88	0.29	0.12-0.48	ns	0.2	0.06-0.36	0.26	0.08-0.76	0.16	0.09-0.42	ns
IFNg+IL-2+TNFa+	5.84	3.34-8.44	6.48	3.93-9.71	5	3.65-8.11	ns	7.22	4.35-8.58	6.1	2.57-8.85	5.61	2.96-8.84	ns
Quad	2.57	1.10-5.12	3.16	1.92-4.51	2.33	1.39-3.44	ns	3	1.39-4.25	2.35	1.51-3.65	2.55	1.11-4.94	ns
No Cytokine CD4	42.88	33.89-55.17	32.39	26.44-44.62	31.63	24.56-40.1	***	44.31	36.3-53.64	35.21	23.21-47.2	33.01	26.12-48.71	ns
CD4+ Total CD107	9.2	5.61-12.8	10.95	7.41-13.7	9.91	6.33-12.58	ns	7.16	4.89-11.2	8.7	5.81-12.15	7.76	6.37-11.83	ns
CD4+ Total IFNg	15.4	8.14-25.4	18.95	12.35-26.78	23.15	12.98-30.83	**	14.3	10.6-20.6	17.75	11.88-27.6	18.95	13.13-32.63	*
CD4+ Total TNFa	42.8	33.7-53.8	52.8	45.7-66.13	58.4	47.85-67.45	ns	42.6	32.75-52	54.35	44.48-65.48	56.9	42.98-63.88	*
CD4+ Total IL-2	37.2	23.6-43.9	43.05	35.83-50.58	44.45	33.98-51.43	**	35.9	26.8-45.6	44.05	35.78-55.03	43.5	30.35-53	ns
CD107+	2.42	1.42-4.08	2.01	0.99-3.59	2.03	1.17-3.48	ns	2.22	1.41-3.76	1.76	1.39-3	1.74	1.12-2.73	ns
IFNg+	0.55	0.26-1.05	0.81	0.41-2.34	0.59	0.3-1.16	ns	0.35	0.22-0.69	0.75	0.36-1.4	0.34	0.23-0.99	ns
IL-2+	8.19	3.11-12.92	5.37	3.42-10.17	5.44	2.53-8.3	**	6.92	3.25-11.34	5.94	3.45-9.19	4.35	2.32-8.5	ns
TNFa+	8.53	5.93-12.24	9.67	6.85-12.29	8.67	6.64-11.56	ns	7.47	4.94-10.11	9.04	5.81-10.57	6.95	4.69-11.01	ns
CD107+IFNg+	0.13	0.04-0.3	0.26	0.12-0.72	0.22	0.07-0.52	ns	0.08	0.04-0.12	0.16	0.07-0.46	0.09	0.04-0.34	ns
CD107+IL-2+	0.14	0.07-0.35	0.16	0.07-0.3	0.13	0.04-0.23	*	0.11	0.04-0.18	0.15	0.05-0.3	0.09	0.04-0.17	ns
CD107+TNFa+	0.54	0.33-0.91	0.54	0.28-0.87	0.58	0.35-0.92	ns	0.43	0.28-0.68	0.47	0.23-0.73	0.45	0.24-0.72	ns
IFNg+IL-2+	0.31	0.12-0.91	0.42	0.17-0.96	0.25	0.07-0.56	ns	0.31	0.13-0.65	0.4	0.17-0.91	0.21	0.09-0.7	ns
IFNg+TNFa+	2.89	1.68-7.19	3.49	1.99-6.75	4.88	2.54-9.07	*	3.47	2.27-5.57	3.61	2.15-6.52	4.54	2.55-8.64	ns
IL-2+TNFa+	15.51	10.72-21.29	21.66	17.22-29.24	22.74	17.62-28.03	***	16.84	10.07-23.49	23.81	16.11-33.07	21.14	15.73-25.17	ns
CD107+IFNg+IL-2+	0.02	0-0.05	0.03	0-0.08	0.01	0-0.04	ns	0.01	0-0.03	0.04	0-0.1	0.01	0-0.05	ns
CD107+IFNg+TNFa+	0.89	0.51-2.33	0.85	0.53-2.17	1.21	0.46-2.39	ns	0.94	0.47-2.31	1.19	0.6-1.99	1.34	0.74-2.66	ns
CD107+IL-2+TNFa+	0.46	0.2-1.05	0.96	0.37-1.81	0.78	0.24-1.48	ns	0.46	0.19-0.76	0.74	0.33-1.4	0.63	0.28-0.95	*
IFNg+IL-2+TNFa+	6.31	3.25-9.1	8.12	5.66-10.64	9.85	6.78-13.91	ns	7.25	4.74-9.67	8.79	6.06-12.64	9.95	6.74-14.86	**
Quad	0.31	0.14-0.8	0.54	0.28-0.86	0.51	0.21-0.86	ns	0.41	0.18-0.84	0.58	0.26-0.98	0.69	0.31-1.12	ns

Table S4. Differential cytokine expression by HIV status. Univariate and multivariate regression analysis was performed on flow data for T cell cytokine expression. Fold (HIV+/HIV-) denotes the ratio of median marker frequency where expression is greater in HIV+ when fold is >1 and greater in HIV- when <1. Significance indicated as ns= no significance, *=p<0.05, **=p<0.01, ***=p<0.001.

Analysis	Timepoint	Cytokine-expressing T cell subtype	Fold change (HIV+/HIV-)	pval
Univariate	Initial	CD8+CD107+IFNg+	2.75	*
		CD8+CD107+IL-2+	1.75	*
		CD4+CD107+IL-2+TNFa+	1	*
		CD4+CD107+IL-2+	1.27	*
		CD4+ Total IFNg+TNFa-	1.3	*
	EOT	Total TNFa+CD8+	1.15	*
		CD8+ Total IFNg+IL-2+	1.14	*
	M3	None	N/A	
Multivariate	Initial	Total CD107+CD8+	1.08	*
		CD8+ Total IFNg+CD107+	1.05	*
		CD4+TNFa+	1.14	*
		CD4+ Total IL-2-TNFa+	1.12	*
		CD4+ Total IFNg+TNFa-	1.3	*
		CD4+CD107+TNFa+	1.26	*
	EOT	CD4+CD107+IL-2+TNFa+	1.3	*
	M3	No Cyokine CD8+	0.72	**
		Total IFNg+CD8+	1.76	**
		Total TNFa+CD8+	1.11	**
		CD8+IFNg+TNFa+	1.07	*
		CD8+CD107+IFNg+TNFa+	1.11	*
		CD8+ Total IFNg-CD107-	0.91	**
		CD8+ Total IFNg-IL-2-	0.88	**
		No Cyokine CD4+	0.96	**
		CD4+ Total TNFa+	1.03	**
		CD4+IL-2+TNFa+	1.08	*
		CD4+ Total IL-2+	1.02	*
		CD4+ Total IFNg-IL-2-	0.95	**
		CD4+ Total IL-2-TNFa-	0.99	**
		CD4+ Total IFNg+IL-2+	1.25	*
		CD4+ Total IFNg-TNFa-	0.96	*
		CD4+ Total IL-2+TNFa+	1.08	*
		CD4+ Total IFNg-IL-2+	1.07	*
		CD4+ Total IFNg+TNFa-	2.4	*
		CD4+ Total IFNg+CD107-	1.15	*

Table S5. Cox proportional hazards regression models for overall survival in T cell subsets. Subsets significantly associated with survival are shown. HR<1 indicates marker expression below the mean expression frequency is associated with lower hazard of death. Subsets containing IL-2 are bolded and highlighted in green (HR<1) and red (HR>1).

**Table 4. Overall survival by T cell marker**					
**Marker**	**Timepoint**	**Cohort**	**HR**	**95% CI**	**Pval**
**CD8+CD107+IL-2+**	**Initial**	**Total**	**0.391**	**0.216-0.709**	**
CD8+CD57- Total CD27+CD28-	Initial	Total	2.76	1.28-5.97	**
CD8+CD57+ Total CCR7+CD45RA-	Initial	Total	0.511	0.278-0.938	*
CD8+CCR7+ Total CD27+CD28-	Initial	Total	2.33	1.08-5.02	*
**CD4+CD107+IFNg+IL-2+**	**Initial**	**HIV-**	**0.251**	**0.0862-0.732**	*
**CD4+IFNg+IL-2+**	**Initial**	**HIV-**	**0.278**	**0.0964-0.8**	*
CD4+IFNg+	Initial	HIV-	0.293	0.1-0.854	*
CD4+CD107+IFNg+	Initial	HIV-	0.302	0.094-0.97	*
CD8+CD57+ Total CCR7+CD45RA-	Initial	HIV-	0.355	0.127-0.991	*
CD8+IFNg+	Initial	HIV-	0.342	0.118-0.994	*
**CD8+CD107+IL-2+TNFa+**	**Initial**	**HIV+**	**0.396**	**0.191-0.825**	*
CD8+CD57- Total CCR7+CD45RA+	Initial	HIV+	0.404	0.183-0.891	*
**CD4+ Total IFNg+IL-2+**	**Initial**	**HIV+**	**2.69**	**1.09-6.61**	*
**CD8+ Total IL-2-TNFa+**	**Initial**	**HIV+**	**2.3**	**1.07-4.95**	*
CD4+ Total IFNg+TNFa-	Initial	HIV+	3	1.04-8.64	*
CD8+CD57- Total CD27+CD28-	Initial	HIV+	2.51	1.01-6.23	*
**CD4+IL-2+**	**EOT**	**Total**	**0.424**	**0.218-0.824**	*
**CD4+Total IL-2-TNFa+**	**EOT**	**Total**	**2.33**	**1.09-4.97**	*
CD8+CD57- Total CD27-CD28-	EOT	Total	2.25	1.02-4.98	*
**CD4+ Total IFNg+IL-2+**	**EOT**	**HIV-**	**0.161**	**0.035-0.741**	*
**CD4+ Total IFNg-IL-2-**	**EOT**	**HIV-**	**5.08**	**1.11-23.2**	*
Total CD8+IFNg+	EOT	HIV-	0.285	0.0857-0.948	*
**CD4+ Total IFNg+IL-2+**	**EOT**	**HIV+**	**6.25**	**1.46-26.7**	*
CD4+ Total IFNg-TNFa-	EOT	HIV+	0.328	0.135-0.797	*
CD4+ Total IFNg-CD107+	EOT	HIV+	0.35	0.151-0.811	*
CD8+ Total IFNg+CD107-	EOT	HIV+	3.29	1.22-8.88	*
CD8+CD57- Total CCR7-CD45RA+	EOT	HIV+	3.27	1.13-9.46	*
CD8+CD57+ Total CCR7+CD45RA-	EOT	HIV+	0.346	0.129-0.925	*
**CD4+IL-2+**	**M3**	**Total**	**0.391**	**0.188-0.812**	*
**CD4+ Total IL-2+TNFa-**	**M3**	**Total**	**0.409**	**0.199-0.84**	*
**CD8+IFNg+IL-2+**	**M3**	**Total**	**0.441**	**0.215-0.906**	*
**CD4+CD107+IL-2+**	**M3**	**Total**	**0.479**	**0.237-0.97**	*
CD4+Quad	M3	Total	0.492	0.243-0.999	*
**CD4+ Total IL-2+TNFa-**	**M3**	**HIV-**	**0.316**	**0.105-0.952**	*
CD8+CD57- Total CD27+CD28-	M3	HIV-	4.72	1.04-21.4	*
**CD4+IL-2+**	**M3**	**HIV+**	**0.26**	**0.0918-0.739**	*
**CD8+ Total IFNg+IL-2-**	**M3**	**HIV+**	**0.3**	**0.116-0.774**	*
CD8+CD57+ Total CD27-CD28+	M3	HIV+	5.29	1.21-23.2	*
CD8+TNFa+	M3	HIV+	3.07	1.01-9.37	*

*p<0.05; **p<0.001
