# Supplementary material for: Patients with Cervical Cancer with and without HIV Infection Have Unique T-cell Activation Profiles despite Similar Survival Outcomes after Chemoradiation
Source: Cancer Res Commun. 2025 Apr 14;5(4):610–20. doi: 10.1158/2767-9764.CRC-24-0364 (PMC11995389; doi:10.1158/2767-9764.CRC-24-0364)

Supplementary Figures

Figure S1. Gating strategies for flow analysis. Example gating strategy on control cells to identify major T cell subsets.

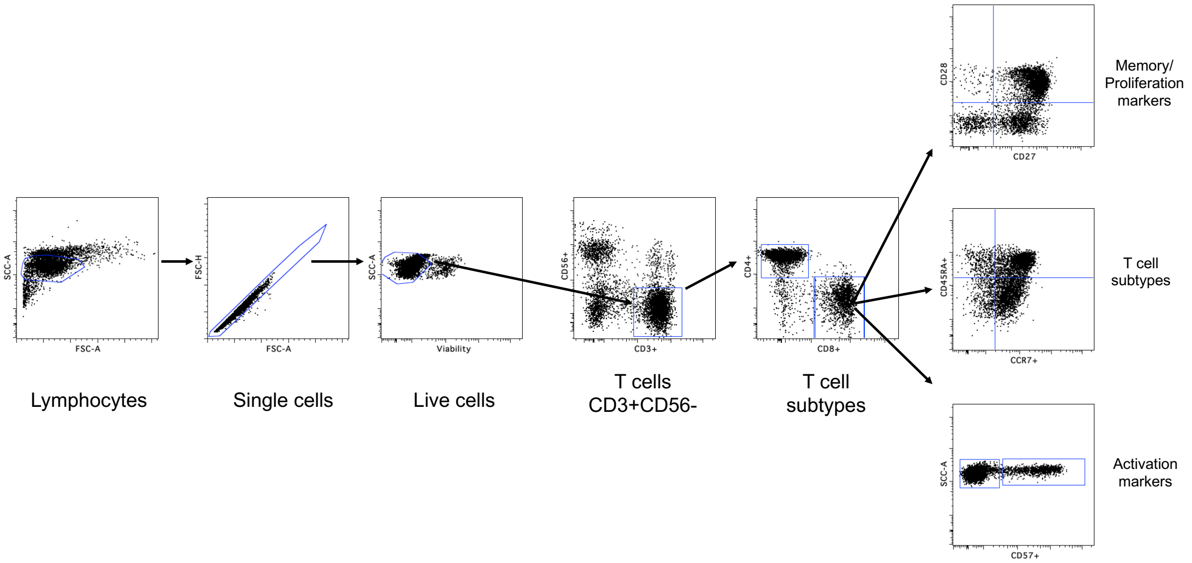

Supplement: Supplementary Figure 1 [file crc-24-0364_supplementary_figure_1_suppsf1.pdf]
