# Supplementary material for: Patients with Cervical Cancer with and without HIV Infection Have Unique T-cell Activation Profiles despite Similar Survival Outcomes after Chemoradiation
Source: Cancer Res Commun. 2025 Apr 14;5(4):610–20. doi: 10.1158/2767-9764.CRC-24-0364 (PMC11995389; doi:10.1158/2767-9764.CRC-24-0364)

Figure S2. Overall survival by HIV status. Kaplan-Meier curve for the total studied cohort comparing patients with (red) and without (blue) HIV infection.

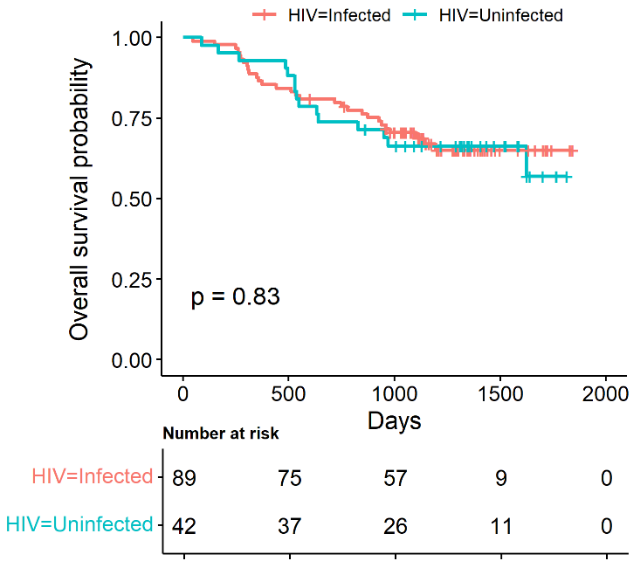

Supplement: Supplementary Figure 2 [file crc-24-0364_supplementary_figure_2_suppsf2.pdf]
