# Supplementary material for: Patients with Cervical Cancer with and without HIV Infection Have Unique T-cell Activation Profiles despite Similar Survival Outcomes after Chemoradiation
Source: Cancer Res Commun. 2025 Apr 14;5(4):610–20. doi: 10.1158/2767-9764.CRC-24-0364 (PMC11995389; doi:10.1158/2767-9764.CRC-24-0364)

Figure S3. Changes in T cell subsets before and after chemoradiation among all patients. A) CD8 T cells and B) CD4 T cells were stratified into four major subsets by two additional markers (CCR7 and CD45RA) to identify naïve (CD45RA+CCR7+), central memory (CD45RA-CCR7+), effector memory (CD45RA-CCR7-), and effector (CD45RA+CCR7-) subsets. Box plots overlay individual patient data points for subset frequencies over three longitudinal study time points (Initial, EOT, M3).

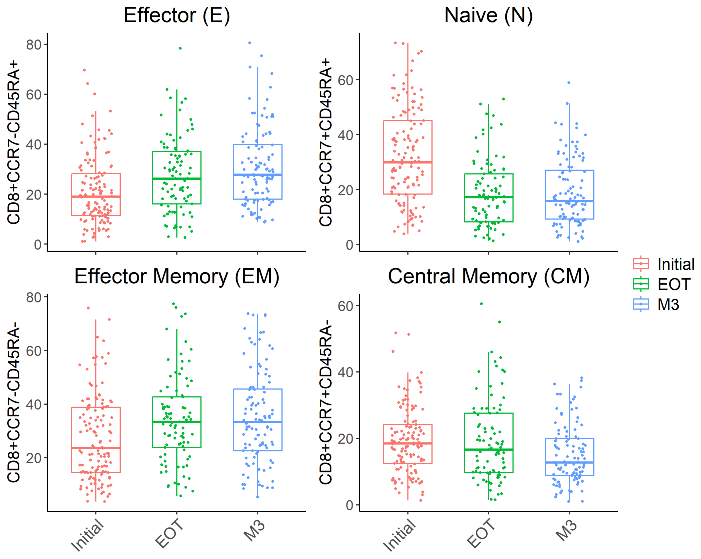

Supplement: Supplementary Figure 3 [file crc-24-0364_supplementary_figure_3_suppsf3.pdf]
