# Supplementary material for: Patients with Cervical Cancer with and without HIV Infection Have Unique T-cell Activation Profiles despite Similar Survival Outcomes after Chemoradiation
Source: Cancer Res Commun. 2025 Apr 14;5(4):610–20. doi: 10.1158/2767-9764.CRC-24-0364 (PMC11995389; doi:10.1158/2767-9764.CRC-24-0364)

Figure S4. Impact of CRT on cytokine expression by T cells from total cohort. Viable CD4 and CD8 T cells from cryopreserved patient PBMCs were stained and analyzed to assess functionality by four primary effector/cytokine markers (CD107a, IFNγ, TNFα, IL-2). Data is displayed as cytokine expression frequencies over various time points using box plots overlaying individual patient samples. Non-matched control samples from "Normal Donor" women are displayed as reference frequency for T cells under presumed "no ongoing activation." A) CD8 T cells, B) CD4 T cells. Significance ($p<0.05$) displayed by asterisk.

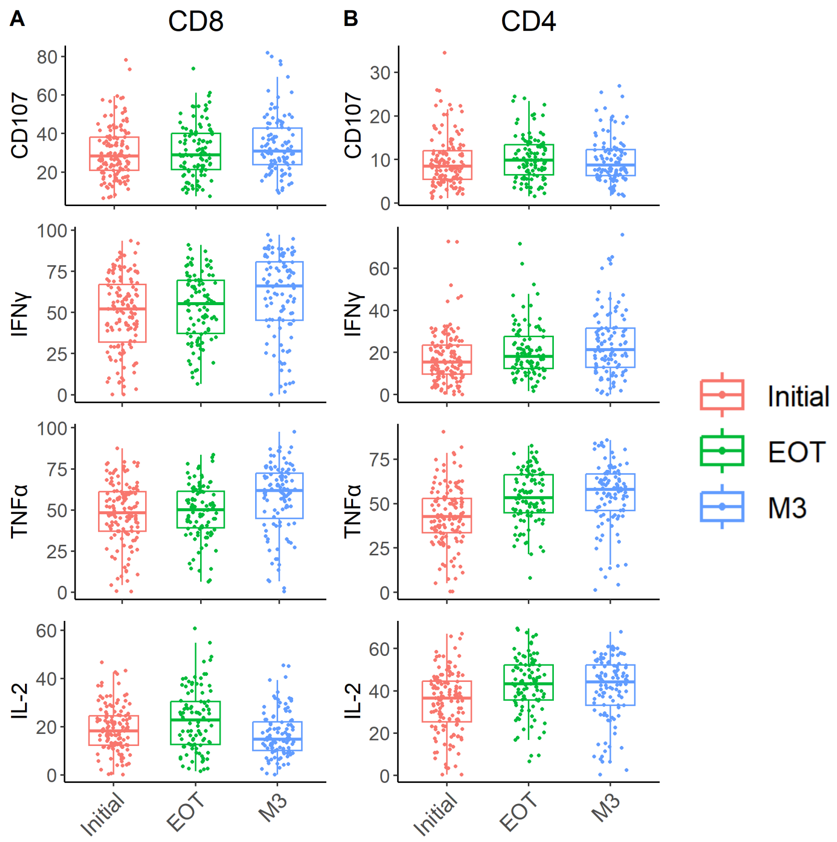

Supplement: Supplementary Figure 4 [file crc-24-0364_supplementary_figure_4_suppsf4.pdf]
